# Insights of bacterial community structure and occurrence of antibiotic resistance and antimicrobial compounds in an urban stream in the megacity of São Paulo, Brazil

**DOI:** 10.1016/j.bjid.2026.105818

**Published:** 2026-04-21

**Authors:** Nazareno Scaccia, Maria Tereza Pepe Razzolini, Gabrielly Lacerda de Aragão, Joyce Vanessa da Silva Fonseca, Nilo José Coelho Duarte, Léonard de Vinci Kanda Kupa, Jonathan Cawettiere Espíndola, Ester Cerdeira Sabino, Anna S. Levin, Silvia Figueiredo Costa

**Affiliations:** a Centres for Antimicrobial Optimisation Network (CAMO-Net) Brazil, Faculdade de Medicina da Universidade de São Paulo (FMUSP), Departamento de Infectologia e Medicina Tropical, São Paulo, SP, Brazil; b Faculdade de Medicina da Universidade de São Paulo (FMUSP), Departamento de Infectologia e Medicina Tropical, São Paulo, SP, Brazil; c Centro Universitário Faculdade de Medicina do ABC (FMABC), Departamento de Patologia, Santo André, SP, Brazil; d Universidade de São Paulo, Faculdade de Saúde Pública (FSP), Departamento de Saúde Ambiental, São Paulo, SP, Brazil; e Hospital das Clínicas da Faculdade de Medicina da Universidade de São Paulo (HC-FMUSP), Divisão de Laboratório Central, São Paulo, SP, Brazil; f Universidade de São Paulo (USP), Centro Internacional de Referência em Reúso de Água (CIRRA), Escola Politécnica, Departamento de Engenheria Hidráulica e Ambiental, São Paulo, SP, Brazil; g Hospital das Clínicas da Faculdade de Medicina da Universidade de São Paulo (HC-FMUSP), São Paulo, SP, Brazil

**Keywords:** Urban stream microbial composition, Antibiotic-resistant bacteria, Antibiotic resistance gene, Antimicrobial residue, Chlorhexidine minimum inhibitory concentrate

## Abstract

This study aimed to investigate the microbial community structure and the presence of Antibiotic-Resistant Bacteria (ARB), Antibiotic Resistance Genes (ARGs), and antimicrobial residues in an urban stream adjacent to an informal settlement in Brazil. Water samples collected during the winter of 2021 (n = 7) were analysed using culture-dependent and -independent methods. Microbial community structure was assessed by 16S rRNA sequencing, while bacterial isolates were characterized by Chlorhexidine Minimum Inhibitory Concentration (CHX-MIC) and whole-genome sequencing. Free carbapenemase ARGs were screened by conventional PCR and selected antimicrobial residues were quantified by UHPLC-MS/MS. The bacterial community was dominated by phyla *Firmicutes, Bacteroidota, Proteobacteria, Campylobacterota, Actinobacteriot*a, and *Patescibacteria*. Abundant (> 5%) families included *Ruminococcaceae, Prevotellaceae, Moraxellaceae*, and *Arcobacteraceae*, associated with faecal contamination and antimicrobial resistance. Urban stream bacterial isolates (31/66) belonged mainly to the genera *Aeromonas, Citrobacter, Escherichia, Serratia, Chryseobacterium, Elizabethkingia* with a high percentage of phenotypic resistance to third-generation cephalosporin and carbapenems. WGS of some isolates (16/31) revealed the co-production of ESBL and carbapenemase ARGs as well as *Inc*-type plasmids, efflux pumps and virulence genes. CHX-MICs ranged from 0.5 to 128 mg/L, with *Klebsiella pneumoniae* and *Serratia marcescens* showing the highest values and harboring *qac* genes and *Inc*HI2 plasmids, respectively. Azithromycin, ceftriaxone, levofloxacin, and chlorhexidine were detected at up to 2.39, 1.18, 0.14, and 0.43 mg/L, respectively. Notably, ceftriaxone-positive water samples also contained ARB and free ARGs (*bla*_NDM_, *bla*_KPC_, *bla*_VIM_). These findings highlight urban streams as reservoirs of ARB, ARGs, and antimicrobial residues, stressing the urgent need for local water treatment to mitigate antimicrobial resistance and public health risks.

## Highlights

• Urban streams are heavily impacted by slum untreated sewage.
• Polluted urban waterways accelerate the dispersion of antibiotic resistance.
• Co-occurrence of antimicrobials, resistant bacteria and resistance genes is alarming.
• *Klebsiella pneumoniae* and *Serratia marcescens* showed high chlorhexidine MICs.
• Stream bacterial community shows fecal and antibiotic bacterial family’s indicator.

## Graphical abstract

**Figure:**
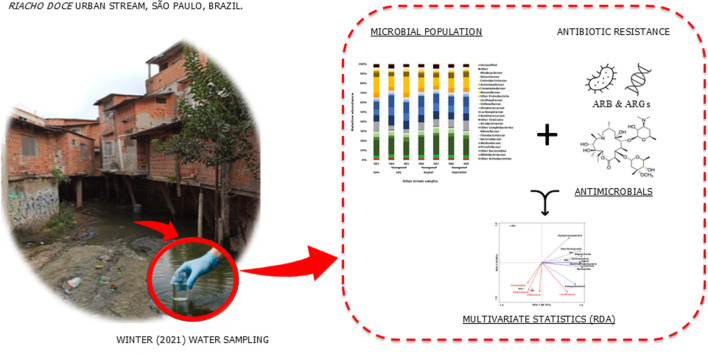

## Introduction

The global rise of Antibiotic Resistance (AR) is considered a serious threat facing public health and well-being worldwide.^1^ Of particular concern, carbapenemase-producing bacteria are listed as critical priority pathogens that pose a major threat to human health due to their resistance to multiple antibiotics.^1^ While clinical settings have been the primary focus of AR, environmental reservoirs, such as aquatic habitats, have been increasingly recognized as critical hotspots for the spread of Antibiotic-Resistant Bacteria (ARB) and Antibiotic Resistance Genes (ARGs).^2^^,^^3^ Anthropogenic levels of antibiotics, biocides (e.g., chlorhexidine) and other contaminants (e.g., heavy metals for instance) have accelerated the proliferation and dissemination of ARB and ARGs across diverse ecosystems.^2^, ^3^, ^4^, ^5^ It is known that the presence of sub-inhibitory concentrations of antibiotics in the environment can exert selective pressure on bacterial communities, promoting resistome mobility and maintenance of ARB.^3^^,^^5^ Biocide compounds, such chlorhexidine, can also promote co-selection for antibiotic resistance in bacteria. This is called indirect selection of ARB when the bacterial community is exposed to non-antibiotics compounds.^4^^,^^5^ It occurs when biocide and ARGs co-exist on the same genetic element, allowing one selective agent to drive resistance to others.

In the megacities of Low- and Middle-Income Countries (LMICs), where population density is high, levels of anthropogenic pollution are elevated and sanitation infrastructures are often inadequate, urban waterways may act as predominant reservoirs of AR, posing risks to human and environmental health.^6^^,^^7^ São Paulo (Brazil), which is the largest metropolitan area of South America, exemplifies the complex interplay between urbanization and environmental contamination caused by the anthropogenic impacts. Indeed, urban water bodies in São Paulo are heavily impacted by untreated domestic, disposal of solid waste and stormwater runoff.^8^, ^9^, ^10^, ^11^ Part of this is driven by unplanned urbanization associated with rapid population growth expansion. This urban phenomenon, combined with the lack of adequate housing policies, rural-urban migration, affordable housing and economic inequality has created the widespread development of irregular urban settlements, locally known as “*favelas*” or slums.^6^^,^^12^ According to the 2022 Census, conducted by the Brazilian Institute of Geography and Statistics, 12.348 informal settlements were identified across 656 Brazilian municipalities, being home to approximately 16.3 million people, which corresponds to 8.1% of the total population.^13^ The city of São Paulo has some of the largest and most populated slums of the country such as *Paraisópolis* and *Heliópolis*, located in the south and southeast area of the city with 58,527 and 55,583 inhabitants, respectively.^13^ These community-driven informal settlements are characterized by irregular urban development models with urban systems gaps such as the lack of sewage systems, irregular waste collection, poor drainage system and absence of basic public services.^12^^,^^13^ It has been estimated that 24.3% of the Brazilian population, which corresponds to approx. to 49 million persons, are not covered by the public sewage infrastructure and untreated wastewater is discharged directly into rivers, streams and the ocean.^13^ Therefore, urban water bodies located within these cities which receive continuous inputs of untreated human waste serve as dynamic conduits for the emergence and circulation of ARB and ARGs. Urban rivers thus function as mixing vessels of ARB, ARGs with other human, clinical and environmental bacteria alongside with residues of antimicrobials and therefore can magnify the dispersion of AR.^3^^,^^6^ Finally, the discharge of untreated wastewater into urban waterways can also lead to the enrichment of these water bodies with pathogenic allochthonous microorganisms that can threaten human health and disrupt the microbial ecological balance of the aquatic ecosystem.^10^^,^^14^

The main objectives of this exploratory study were to i) Characterize the bacterial community composition; ii) Investigate the presence of AR, particularly carbapenemase-producing bacteria; iii) Explore the presence of free carbapenemase-encoding genes; and iv) Evaluate antimicrobial concentrations in an urban stream that crosses a *favela* in the city of São Paulo. Ultimately, the likely interaction between the microbial population and antimicrobial residues was also explored.

## Material and methods

### Setting

**Fig. 1:**
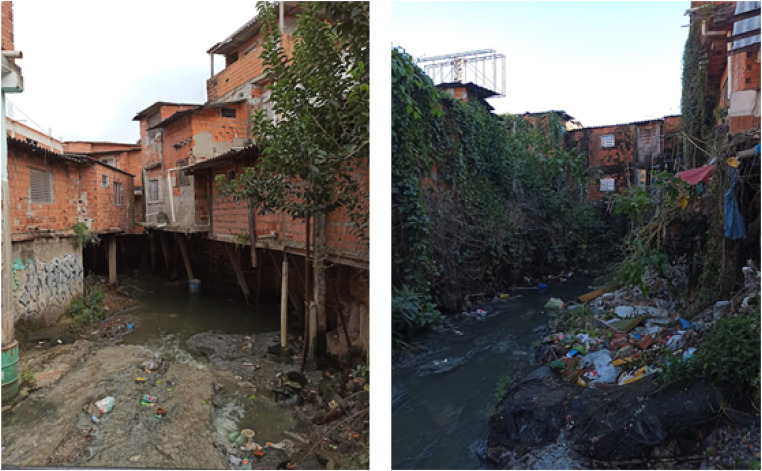
*Riacho Doce* stream crossing the *São Remo* informal settlement within São Paulo.

The water samples used for this study were collected from an urban stream called “*Riacho Doce*” located within the *São Remo* community, in the western administrative area of São Paulo, next to the main campus of the University of São Paulo. According to the 2022 census, the informal settlement of *São Remo* has an estimated population of 7979 inhabitants.^13^ The community is linked to the public potable water supply, however, there is no sewage system. Thus, untreated wastewater is directly discharged into the “*Riacho Doce*” stream. We collected water samples from this stream (Fig. 1). One hospital, *Hospital Universitário da USP (HU/USP)*, and a primary health care unit that serves the community are located near this area.

### Collection of water samples

The sampling campaign was carried out during the winter season (June ‒ September 2021). The samples were collected twice per month (n = 7), except in June, when samples were collected once. A volume of one litre of water was collected in a sterilized wide-mouth polypropylene bottle and transported to the laboratory in a cool box filled with ice where it was processed within two hours.

From each water sample, aliquots of 1, 100–150 and 10 mL were used for cultivable, Total Community DNA (TC-DNA) extraction and antibiotic and chlorhexidine residues quantification, respectively. Samples for bacterial enumeration and TC-DNA extraction were immediately processed whilst water aliquots for antibiotic and chlorhexidine residues detection were rapidly frozen and stored at −80 °C until further analysis.

### Total community DNA extraction

For extraction of the TC-DNA, 100 to 150 mL of freshly collected water samples were filtered through a 0.22 µm pore-size cellulose membrane (Millipore, Merck) by filtration unit, and the membranes were then stored at −80 °C. Prior to TC-DNA extraction, membranes were aseptically cut into four pieces and placed into 2 mL Eppendorf tubes and subsequently, the PowerSoil DNA isolation kit (MO BIO Laboratories) was used according to manufacturer’s instructions. Beside the bacterial community analysis, the TC-DNA extracts were also used to survey the presence of carbapenemase genes *bla*_NDM_, *bla*_KPC_, *bla*_SPM_, *bla*_IMP_, *bla*_VIM_, *bla*_OXA-23_ and *bla*_OXA-48_. These analyses were based on conventional PCR as described below. Additional information concerning sampling dates, volumes filtered, and TC-DNA concentrations are reported in Table S1.

### Bacterial community analysis

The bacterial community composition was analysed based on the V4 region of the 16S rRNA gene as previously described.^15^ The sequencing was carried out with the Ion PGM™ Sequencing 400 Kit in an Ion Torrent™ Personal Genome Machine (Thermo Fisher) using an Ion 318™ chip kit v2 (Thermo Fisher). All samples were sequenced once. Demultiplexed raw reads were processed using the latest version of the Ion Torrent server (version 5.0.4). The resulting reads were processed and analysed by Quantitative Insights into Microbial Ecology (QIIME2) (www.qiime2.org; version 2019.10). Sequences shorter than 200 bp and with average quality scores lower than 25 were eliminated. Sequences were filtered and merged and, chimeric reads were removed by the DADA2 software package enclosed in QIIME2. Taxonomy was assigned to the Amplicon Sequence Variants (ASVs) using a pre-trained Naive Bayes classifier trained on the ARB SILVA database (www.arb-silva.de; release 138) as previously described.^15^ A total of 674,403 reads (ranging from 46,793 to 148,852 reads per sample) (Table S1) and 3402 ASVs (ranging from 531 to 1040 per sample) were obtained from the 7 datasets. Rarefaction curves for all samples reached a plateau, indicating that the sequencing depth was sufficient to capture bacterial community diversity and richness.

### Isolation and characterization of antibiotic-resistant bacteria from stream water

In this exploratory study, we aimed to investigate the potential presence of clinically relevant groups such as ESBL-producing Enterobacteriaceae and Vancomycin-Resistant Enterococci (VRE). Briefly, ten-fold serial dilutions of two water Samples from July (SR5) and August (SR6) were prepared in sterile saline solution (0.85% [w/v] NaCl) and 100 µL of different dilutions were plated directly onto culture media chromID™ ESBL and CHROMID® VRE (BioMerieux) and incubated for 24 h at 37 °C. Different colonies from each medium with a specific colour of the targeted ARB, according to the instructions of the manufacturer, were picked up and streaked onto MacConkey agar medium. The purified cultures were preserved in Brain Heart Infusion medium (Oxoid) supplemented with 20% (v/v) of glycerol and stored at −80 °C. Out of 66 isolates obtained, a subset (n = 31) of bacterial strains were selected based on phenotypic production of carbapenemases and the presence of targeted ARGs (see next section). Bacterial taxonomic classification was assigned by MALDI-TOF MS (Bruker) and 16S rRNA gene sequence analysis. The selected isolates were tested for susceptibility to several antibiotic classes: β-lactam, such as Aztreonam (ATM, 30 μg), Amoxicillin-Clavulanate (AMC, 20/10 μg) as well as third-generation cephalosporin (Ceftriaxone [CRO, 30 μg], Ceftazidime [CAZ, 30 μg], Cefotaxime [CTX, 30 μg]) and carbapenems (Ertapenem [ERT, 10 μg], Meropenem [MPM, 10 μg], Imipenem [IPM, 10 μg]; quinolone (Levofloxacin [LEV, 5 μg]) and aminoglycoside (Gentamicin [GEN 10 μg]) based on the disk diffusion method, according to the guidelines of the Clinical and Laboratory Standards Institute (CLSI).^16^ The antibiotic susceptibility of these isolates was also evaluated by phenotypic test of carbapenemase activity using the Carbapenembac® (Probac do Brazil) assay. The Minimum Inhibitory Concentration (MIC) values for chlorhexidine were also determined by the agar dilution test. For that, serial logarithmic concentrations of chlorhexidine were added in Mueller-Hinton agar culture media distributed onto individual Petri dishes. The tested chlorhexidine concentrations ranged between 0 and 256 mg/L and Klebsiella pneumoniae ATCC 13883 (chlorhexidine MIC of 16 mg/L) and *Escherichia coli* ATCC 25922 (chlorhexidine MIC of 2 mg/L) were used as controls.^17^

### DNA extraction from the bacterial isolates

To screen ARGs, genomic DNA was obtained using the boiling method. Briefly, the isolates were grown overnight at 37 °C on MacConkey agar medium and successively, a few colonies were picked up and diluted in 500 μL TE buffer. The bacterial suspensions were centrifuged at 7000 rpm for 2-min, and the pellet was resuspended in a new TE buffer. The suspension was incubated at 100 °C for 10-mins and then rapidly cooled in ice and centrifuged as indicated above. Finally, the supernatant was collected and stored at −20 °C before being used as a template for amplification reactions.

For whole genome sequencing analysis, the DNA was extracted by QIAamp DNA Mini Kit (QIAGEN, Hilden, Germany) following the instruction of the manufacturer. The concentration of the DNA extracts obtained were determined through 260/280 nm absorbance measures using NanoDrop spectrophotometer (Thermo Scientific, Waltham, USA) and stored at −20 °C for further analysis.

### Screening of antibiotic resistance genes in the isolated bacteria

All the bacterial isolates obtained were screened for the presence of genes conferring resistance to carbapenems such as: *bla*_NDM_, *bla*_KPC_, *bla*_SPM_, *bla*_IMP_, *bla*_VIM_, *bla*_OXA-23_ and *bla*_OXA-48_. PCR primer sequences (Síntese Biotecnologia LTDA), concentrations, amplicon size and annealing temperatures are shown in Table 1. The PCR conditions were as follows: denaturation at 94 °C for 5-min for all the ARGs, followed by 35-, 33-, 45- and 30-cycles for *bla*_NDM_ and *bla*_KPC_, *bla*_IMP_ and *bla*_SPM_, *bla*_VIM_, *bla*_OXA-23_ and *bla*_OXA-48,_ respectively, consisting of denaturation at 94 °C for 40 s for all the genes; annealing as reported in Table 1 for 40 s; extension at 72 °C for 40 s and 50 s for *bla*_NDM_, *bla*_KPC_ and *bla*_IMP_, *bla*_SPM_, *bla*_VIM_, *bla*_OXA-23_, *bla*_OXA-48,_ respectively, and finalized by extension at 72 °C for 6-min. For all the PCR reactions, positive internal controls as previously reported^18^^,^^19^ were used. The PCR products were analysed by electrophoresis in 1.5% agarose gel, stained with SYBR Safe DNA Gel Stain (Invitrogen), and visualized by UV transillumination. The obtained PCR amplicons were purified with the GFX™ PCR DNA and Gel Band Purification Kit (Merck) and subjected to Sanger sequencing (ABI 3730 DNA Analyzer) at the Faculty of Medicine of the University of São Paulo to confirm the target amplified genes.

**Table 1:** Oligonucleotides DNA sequences used for the PCR to detect carbapenemase genes.

**Target genes**	**Primer**	**Sequence (5′ to 3′)**	**Concentration (µM)**	**Amplicon size (bp)**	**Annealing temp (**°**C)**	**References**
*bla*_KPC_	KPC F3	TGGGCAGTCGGAGACAA	0.1	192	58	20
	KPC B3	GTTGACGCCCAATCCCTC	0.1			
*bla*_NDM_	NDM1 F3	GCTTGCCCCGCAAGAG	0.2	182	58	20
	NDM1 B3	AGCCACCAAAAGCGATGTC	0.2			
*bla*_IMP_	IMP F3	GCAGAGTCTTTGCCAGAT	0.2	243	54	20
	IMP B3	GTCGCTATGAAAATGAGAGG	0.2			
*bla*_VIM_	VIM F3	CCTGTAACGCGTGCAGTC	0.4	218	60	20
	VIM B3	GCAGCACCAGGATAGAAGAG	0.4			
*bla*_SPM_	SPM F1	CTAAATCGAGAGCCCTGCTTG	0.2	798	52	21
	SPM R1	CCTTTTCCGCGACCTTGATC	0.2			
*bla*_OXA-23_	OXA23 F3	GGGCGAGAAAAGGTCATT	0.4	189	54	20
	OXA23 B3	ACCAACCAGAAATTATCAACC	0.4			
*bla*_OXA-48_	OXA48 F3	AATAGCTTGATCGCCCTC	0.4	190	49	20
	OXA48 B3	CCATAATCGAARGCRTGYAGC^a^	0.4			

a The degenerate base is denoted by R (A or T) and Y (C or T). All PCR reactions were conducted in singleplex.

### Whole genome sequencing

The DNA extracts of the isolates were quantified using the Qubit High Sensitivity kit and the Qubit equipment (Thermo Fisher). Following the manufacturer's protocols, the samples were diluted to start the protocol with 50 ng of DNA, which was fragmented to 400 bp using the Covaris™ S2 System equipment (Covaris, USA). Using the reagents from the Ion Xpress™ Plus Fragment Library Kit (ThermoFisher) the fragmented samples was proceeded to the steps of repairing the ends, binding of adapters and binding of barcodes for further purification using Agencourt™ AMPure™ XP (Beckman Coulter, USA). At the end of library construction, fragments of approximately 400pb were selected using the E-Gel™ SizeSelect™ II Agarose Gel, 2% kits and the E-Gel™ equipment (ThermoFisher, USA). The sequencing libraries of each sample were quantified by qPCR using the Ion Library TaqMan™ Quantitation Kit in the QuantStudio equipment (ThermoFisher). Samples were diluted to a final concentration of 40 pM and then pooled to form the sequencing pool. The sequencing pool was inserted into the sample compartment of the Ion PGM HI-Q View Chef 400 kit, which was inserted into the Ion Chef instrument (Thermo Fisher) for PCR reactions in oil and water, purification and feeding of the 318 V2 BC chip (Thermo Fisher). The quality of the files generated in the sequencing was evaluated by FastQC v. 0.11.3 and Trimmomatic v.0.33. The genome assembly was performed using the bacterial Bioinformatics Resource PATRIC (PathoSystems Resource Integration Center) (www.bv-brc.org). All genomes were aligned with the reference genome, available on the NCBI (National Center for Biotechnology Information) website (www.ncbi.nlm.nih.gov). The bacterial species identification, acquired antibiotic resistance genes as well as virulence genes were searched using the freely available web-services provided by the Centre for Genomic Epidemiology (CGE) (www.genomicepidemiology.org) such as SpeciesFinder-2.0, ResFinder-4.5.0 and VirulenceFinder-2.0, respectively. Furthermore, the genomes were inspected for the presence of ARGs using the CARD (Comprehensive Antibiotic Resistance Database) platform (card.mcmaster.ca/). The presence of plasmids was also performed using the PlasmidFinder-2.0 tool provided by the CGE. The Type Sequences (ST) of the isolates were verified by MLST-2.0 CGE web-services and the public databases for molecular typing and microbial genome diversity (PubMLST) (pubmlst.org/).

### Pharmaceutical compounds analysis

Concentrations of chlorhexidine (antiseptic) ceftriaxone and meropenem (beta-lactams), azithromycin (macrolide), levofloxacin (fluoroquinolone) and gentamicin (aminoglycoside) were determined by Ultra-High-Performance Liquid Chromatography coupled with Mass Spectrometer (UHPLC-MS/MS) as previously reported.^15^ Briefly, a solution composed of 100 μL of filtered water samples, 55 μL of internal standard working solution, 45 μL of surrogate working solution and 900 μL of pure water were prepared and injected into the UHPLC Thermo Scientific Ultimate 3000 system coupled with a TSQ Altis™ triple quadrupole mass spectrometer. In parallel, calibration curves and quality controls were also prepared similarly. Accucore C18 2.6 μm (2.1 × 100 mm) column set at 30 °C with a linear gradient of mobile phase at 0.3 mL/min of flow was used for the separation of the analytes. For these chemicals, the Limits of Quantifications (LOQ) were 0.11 µg/L for azithromycin, chlorhexidine, levofloxacin and meropenem; 0.44 µg/L for ceftriaxone and, 22.22 µg/L for gentamicin.^15^ Moreover, although other compounds may also contribute to the overall selective pressure, these compounds were selected for investigation due to their use during the COVID-19 pandemic and their previous identification in the nearby region.^15^

### Statistical analysis

The bacterial community composition was expressed as the relative abundance of reads of a specific bacterial group per total reads number. To examine how the abundance of specific bacterial taxa changed throughout the period of study, one-way analysis of variance (ANOVA) and Tukey's post hoc test were used for determination of statistically significant differences (p < 0.05) (“multcomp” and “stats”, *R* package, version 4.4.2). Additionally, multivariate statistical analyses such as Redundancy Analysis (RDA) at class level (with relative abundance > 1%) to explore whether antimicrobial residues could influence the bacterial community structure was also carried out using the software Canoco version 5.01 as previously described.^15^ Meteorological conditions such as temperature and precipitation were also collected (Table S1).

## Results

### Microbial community analysis

**Fig. 2:**
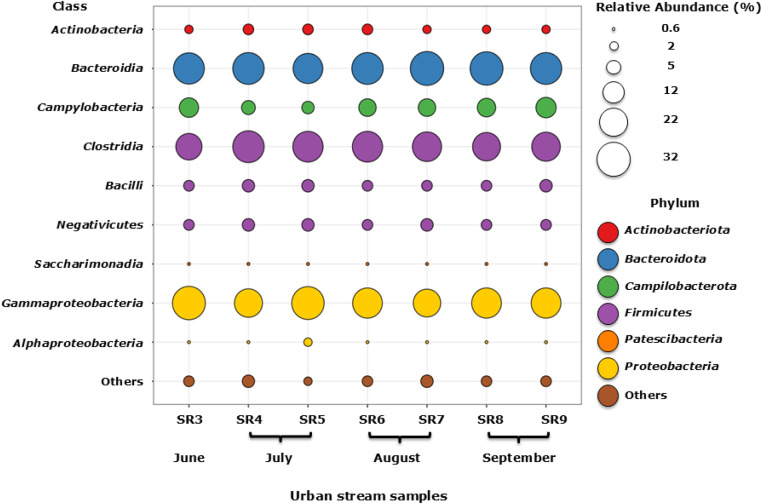
Relative abundance of predominant phyla groups from urban stream samples collected during the winter season (June – September) 2021. Only major taxa (> 2% total abundance) were included. Phyla with abundances < 2% are designated as “Others”.

The bacterial composition of the urban stream was dominated by members of the class *Bacilli* (2.5%‒4.2%), *Clostridia* (18.8%‒28.4%) and *Negativicutes* (2.7%‒3.8%) under the *Firmicutes* phylum; *Bacteroidia* (25.2%‒32%) within the *Bacteroidota* phylum; *Alphaproteobacteria* (0.7%‒1.3%) and *Gammaproteobacteria* (20.9%‒30.7%) belonging to the *Proteobacteria* phylum; *Campylobacteria* (3.8%‒10.5%) under the *Campilobacterota* phylum; *Actinobacteria* (2.1%‒2.9%) within the *Actinobacteriota* phylum and *Saccharimonadia* (0.7%‒1.4%) that belong to the *Patescibacteria* phylum (Fig. 2). Within these bacterial taxa, the predominant bacterial families such as *Lachnospiraceae* (8.2%‒12,9%), *Ruminococcaceae* (7%‒8.4%), *Oscillospiraceae* (1.3%‒2%), *Streptococcaceae* (1%‒2%) (*Firmicutes* phylum), *Prevotellaceae* (16.1%‒19.8%), *Bacteroidaceae* (2.6%‒4.4%), *Weeksellaceae* (1.9%‒4.7%), (*Bacteroidota* phylum), *Moraxellaceae* (8.7%‒17.4%), *Comamonadaceae* (3.9%‒5.3%), *Rhodocyclaceae* (1.2%‒2.6%), *Neisseriaceae* (1.1%‒2.4%), *Enterobacteriaceae* (0.8%‒2.4%), *Aeromonadaceae* (0.7%‒1.9%) (*Proteobacteria* phylum), *Arcobacteraceae* (3.7%‒10.2%) (*Campilobacterota* phylum) and *Bifidobacteriaceae* (0.9%‒1.7%) (*Actinobacteriota* phylum) were observed (Fig. S1). Statistically significant variations (p < 0.05) were observed throughout the study period for the phyla *Firmicutes, Campilobacterota* and *Actinobacteriota* (Table S2). Specifically, the phyla *Firmicutes* and *Actinobacteriota* decreased from July (34.9 ± 2.3 and 3.4 ± 0.3, respectively) until September (28.9 ± 1.5 and 2.6 ± 0.1, respectively), while *Campilobacterota* increased from the beginning (4.4 ± 0.8) until the end (9.6 ± 1.3) of the winter season (Table S2). The main bacterial families responsible for this variation under these bacterial phyla were *Ruminococcaceae* and *Veillonellaceae* for *Firmicutes* and *Arcobacteraceae* for *Campilobacterota.* Other bacterial families that showed a statistically significant increase (p < 0.05) over the winter were *Flavobacteriaceae* (*Bacteroidota* phylum), *Comamonadaceae, Aeromonadaceae* and *Rhodocyclaceae* (*Proteobacteria* phylum) (Table S2). Temperature and precipitation conditions during the winter season were stable and probably did not explain the changes in microbial population (Table S1).

### Characterization of the stream bacterial isolates

A total of 66 bacterial isolates were recovered only from water samples collected in July and August 2021. All the strains were screened for the presence of ARGs such as *bla*_NDM_, *bla*_KPC_, *bla*_SPM_, *bla*_IMP_, *bla*_VIM_, *bla*_OXA-23_ and *bla*_OXA-48_ by PCR. Thirty-one (47%) bacterial strains were only positive for ARGs *bla*_KPC_ (20%) and *bla*_VIM_ (23%) (Table 2). All the bacterial strains were identified as belonging to 17 bacterial species under the *Proteobacteria* (*Gamma-* and *Beta-proteobacteria* classes) and *Bacteroidota* (*Flavobacteriia* class) phyla. Specifically, the *Gammaproteobacteria* were identified as members of the genera *Aeromonas, Citrobacter, Escherichia, Enterobacter, Klebsiella, Kluyvera, Pseudomonas* and *Serratia* whilst, under the *Betaproteobacteria,* only the genus *Comamonas* were found (Table 2). With regard to the *Bacteroidota* phylum*,* members of the genera *Chryseobacterium* and *Elizabethkingia* were identified. The phenotypic test for carbapenemase-production, was positive in 64.5% (20/31). Antibiotic susceptibility was 87% for levofloxacin; 74% for gentamicin; 61% for imipenem; 61% for meropenem; and 59% for ceftazidime. On the other hand, resistance was 77% to cefotaxime; 68% to amoxicillin/clavulanic acid; 58% to aztreonam; and 55% to aztreonam.

**Table 2:** Bacterial strains isolated from “*Riacho Doce*” stream (City of São Paulo, Brazil). Species identification, antimicrobial susceptility, presence of Antimicrobial Resistance Genes (ARGs), and phenotypic test results for Carbapenemases (CARB) within the isolates identified, is listed.

The bacterial isolates were recovered from the urban stream “*Riacho Doce*” collected in July (SR5) and August (SR6) 2021. CAZ, ceftazidime; CTX, Cefotaxime; CRO, Ceftriaxone; ATM, Aztreonam; AMC, Amoxicillin/Clavulanic Acid; IPM, imipenem; ERT, Ertapenem; MPM, Meropenem; LEV, Levofloxacin and GEN, Gentamicin. Only the ARGs detected were reported: +, positive; -, negative. Among the isolates, ARGs blaNDM, blaSPM, blaIMP, blaOXA-23 and blaOXA-48 were not found.

Antibiotic susceptibility: black, resistant; gray, intermediate; white, susceptible.

^a^In bold, bacterial isolates selected for WGS analysis.

^b^CARBAPENEMBAC® (CARB), phenotypic test for rapid detection of metallo-carbapenemase enzymes.

^c^MIC CHX, minimum inhibitory concentration for chlorhexidine.

^⁎^This isolate was initially selected because it was positive for *bla_S_*_PM_. However, the PCR amplicon sequencing did not confirm this gene.

Also, we encountered a high percentage of intermediate susceptibility to Ceftriaxone (CRO, 51.6%) and Aztreonam (ATM, 32.2%) (Table 2). Six out of thirty-one (19.3%) isolates showed resistance to different classes of antibiotics, with one isolate, (SR6-R19) *Serratia marcescens* exhibiting resistance to all the antibiotic classes tested (β-lactam, quinolone, and aminoglycoside) (Table 2).

The CHX-MIC values were determined for the bacterial isolates harbouring the ARGs and they have shown MICs values that ranged from 0.5 to 128 mg/L (Table 2). *Serratia marcescens* and *Klebsiella pneumoniae* had the highest MIC; 128 and 64 mg/L, respectively. *Elizabethkingia anophelis* also showed high MIC of 32 and 64 mg/L. *Comamonas jiangduensis* had the lowest CHX-MIC.

### Whole genome sequencing analysis

Sixteen out of thirty-one bacterial isolates harbouring gene *bla*_KPC_ and/or closely related to human pathogens were sequenced. Whole genome sequencing analysis revealed a broad diversity of ARGs, plasmids and efflux pumps conferring resistance to different classes of antibiotics. *Aeromonas caviae* (*Gammaproteobacteria* class) harbored various ARGs such as *aac(*6′)-Ib-cr, *aac*(3)-Iid, *bla*_KPC-2_, *bla*_TEM_; *bla*_OXA-504_, *mph*A/E and *sul*1 confering resistance to antibiotic classes such aminoglycosides, beta-lactams, macrolides and sulfonamides, respectively. The presence of plasmids (*Inc*P and *Inc*Q) and virulence genes (*clp*K2) were also found (Table S3). The bacterial species belonging to the *Enterobacteriaceae* family (*Gammaproteobacteria* class) displayed a wide array of ARGs conferring resistance to beta-lactams [ARGs *bla*_TEM_ (75%, 6/8), *bla*_CTX_ (37.5%, 3/8) and *bla*_OXA_ (37.5%, 3/8)] and to sulfonamides [ARGs *sul*1 (62.5%, 5/8) and *sul*2 (37.5, 3/8)] (Table S3). The *Enterobacteriaceae* strains also presented ARGs such us *aph*(3′)-Ia, *aph*(3′')-Ib, *aph*(6)-Id and *aad*A5 in *E. coli; aad*A1 and *aac*(6′)-Ib3 in *K. pneumoniae; aac*(3)-IIa, *aa*c(6′)-Ic, *aad*A1, *aph*(3′')-Ib, *aph*(3′)-VIa, *aph*(6)-Id in *S. marcescens; rmt*G and *aac*(6′)-Ib3 in *E. cloacae* that confer resistance to aminoglycosides. In *E. coli* and *S. marcescens,* ARGs *qnr* (*qnr*S1, *qnr*S2, *qnr*B1), *tet* (*tet*A, *tet*B) and *dfr* (*dfr*A1, *dfr*A7, *dfr*A14 and *dfr*A17) responsible for resistance to quinolones, tetracyclines and diaminopyrimidines, respectively, were also detected. Further, resistance to polypeptides (ARGs *arn*T and *ept*B) and fosfomycin (ARGs *fos*A5 and *fos*A6) in *K. pneumoniae* was observed (Table S3). *E. coli, K. pneumoniae* and *C. amalonaticus* also harboured several efflux pump genes such as *emr*R as well as *bae*R, *hns, mar*A and *msb*A belonging to the Major Facilitator Superfamily (MFS), Resistance-Nodulation-cell Division (RND), and ATP-Binding Cassette (ABC) family, respectively. All the isolates of *S. marcescens* displayed the *crp* gene within the RND family whilst, *E. cloacae* did not show efflux pumps genes. The plasmids content in *Enterobacteriaceae* family was represented by the Inc- (FIB, FII, HI2, HI2A, P, Q, R, X and Y) and Col- (*Col*440I, *Col*440II and pHAD2) type plasmids (Table S3). Among the Inc-type plasmids, the *Inc*FIB and *Inc*Q were the most frequent (62.5%, 5/8) being present in *E. coli, K. pneumoniae, S. marcescens, C. amalonaticus* and *E. coli, A. caviae, S. marcescens*, followed by *Inc*HI2, *Inc*HI2A and *Inc*P. The *Inc*HI2 and *Inc*HI2A were found in *S. marcescens* and *E. coli.* The Col-type plasmids were found in *K. pneumoniae, S. marcescens* and *C. amalonaticus* (37.5%, 3/8). Furthermore, a wide diversity of virulence genes in *E. coli, K. pneumoniae* and *C. amalonaticus*, was also detected. The two *E. coli* strains harbour a wider array of virulence genes such as *asl*A, *csg*A, *fde*C, *fim*H, *gad, hly*E, *hly*A, *iss, nlp*I, *ter*C, *yeh*A/B/C/D whilst, *K. pneumoniae* and *C. amalonaticus* possess other virulence determinants *iut*A, *mrk*A, *nlp*I, *tra*T, *anr* and *clp* (Table S3).

Within the *Betaproteobacteria* class, one isolate among the *Comamonas jiangduensis* species harboured the ARGs *aad*A1, *mph*E, *msr*E and *sul*1 that confer resistance to aminoglycosides, macrolides and sulfonamides, respectively. The presence of plasmids and virulence genes in *Comamonas jiangduensis* were not detected. Similarly, the species *Chryseobacterium hispalense,* under the phylum *Bacteroidota*, did not show the presence of either ARGs, plasmids or virulence genes (Table S3). Additionally, the gene *qac*E and *qac*Edelta1, responsible for the resistance to disinfectants and antiseptics, were found in *E. coli, K. pneumoniae, A. caviae, E. cloacae, Citrobacter amalonaticus* and *Comamonas jiangduensis*.

### Occurrence of antimicrobial residues and ARGs in the stream

The antibiotic azithromycin was found in all the water samples with concentrations ranging from 0.67 to 1.18 μg/L. Ceftriaxone was the second most abundant antibiotic found followed by levofloxacin and chlorexidine (Table 3). Gentamicin and meropenem were detected in August and September, however, their concentration was under the LOQ (Tables 3). The DNA extracts of all the water samples were also screened for the presence of carbapenem resistance genes *bla*_NDM_, *bla*_KPC_, *bla*_SPM_, *bla*_IMP_, *bla*_VIM_, *bla*_OXA-23_ and *bla*_OXA-48_ by conventional PCR. Only three ARGs were detected. Specifically, *bla*_KPC_ and *bla*_VIM_ were found in all the samples, whereas ARG *bla*_NDM_ was detected in August and September (Table 3). Since the genes were not quantified, it was not possible to perform a direct correlation analysis between ARGs and the detected antimicrobial residues.

**Table 3:** Concentration of antimicrobial residues (μg/L) and ARGs in the urban stream “*Riacho Doce*” during July–September 2021 (São Paulo, Brazil).

**Chemical group/ ARGs**	**Antibiotic/ resistance target**	**PNEC (μg/L)**	**SR5 Jul**	**SR6 Aug**	**SR7 Aug**	**SR8 Sept**	**SR9 Sept**
Aminoglycoside	Gentamicin	1	0.00	0.00	<LOQ	<LOQ	<LOQ
Carbapenems	Meropenem	0.064	0.00	0.00	<LOQ	<LOQ	0.00
Cephalosporins	**Ceftriaxone**	**0032**	0.00	**2.01**	**1.72**	0.00	**2.39**
Fluoroquinolones	**Levofloxacin**	**0.25**	**0.14**	<LOQ	<LOQ	<LOQ	<LOQ
Macrolide	**Azithromycin**	**0.25**	**0.67**	**1.09**	**0.89**	**0.51**	**1.18**
Antiseptic	**Chlorhexidine**	n.f.	0.00	0.00	0.00	0.00	**0.43**
***bla*_KPC_**	Carbapenem	-	+	+	+	+	+
***bla*_VIM_**		-	+	+	+	+	+
***bla*_NDM_**		-	n.f	+	+	+	+

Samples with concentrations below the Limit of Detection (LOD) were assigned “zero”. <LOQ, indicates values below the limit of quantification. ARGs detected: +, positive; -, negative. The aliquots of the samples SR3 and SR4 were damaged and therefore were excluded from the chemical analysis. PNEC, Predicted No Effect Concentrations (PNECs) values for resistance selection (see discussion section for details). n.f., not found. LOQ values for the antimicrobials (µg/L) were as follows: 0.11 for azithromycin, chlorhexidine, levofloxacin and meropenem; 0.44 for ceftriaxone and, 22.22 for gentamicin.^15^

### Correlation analysis between antimicrobials and the bacteria community structure

**Fig. 3:**
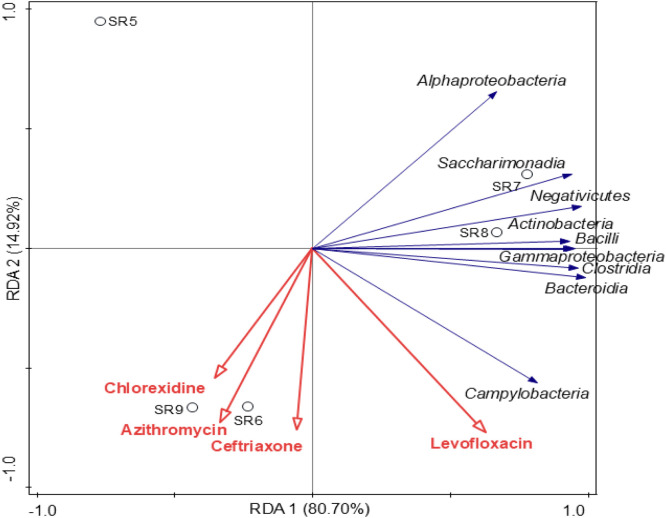
Redundancy analysis of the bacterial classes (with relative abundance > 1%) with chlorhexidine, azithromycin, ceftriaxone, and levofloxacin (in concentrations above the LOQ).

Redundancy Analysis (RDA) was carried out to explore the relationship between bacterial community composition and chlorhexidine, azithromycin, ceftriaxone, and levofloxacin (Fig. 3). Chlorhexidine, azithromycin and ceftriaxone were positively correlated with each other and negatively correlated with *Alphaproteobacteria, Saccharimonadia, Negativicutes,* and *Actinobacteria.*

Levofloxacin instead, was positively correlated with *Campylobacteria, Bacteroidia,* and *Clostridia.* These correlations were not statistically significant suggesting that these antimicrobial residues did not affect the variation of the bacterial population. When evaluating bacterial families’ similar results were observed. However, the presence of these pharmaceutical contaminants at such low levels and the small number of samples, may explain the lack of statistically significant correlations in the RDA analysis despite the observed trends.

## Discussion

In this study, the bacterial community structure of the urban stream “*Riacho Doc*e” (São Paulo, Brazil) was characterized, revealing bacterial families associated with faecal contamination and antibiotic resistance. The presence of carbapenem-resistant bacteria, free carbapenemase genes and residues of azithromycin and ceftriaxone were also found. Although pollutantion in Brazilian rivers has been widely reported, yet comprehensive assessments of the microbial population and AR remain limited.^22^, ^23^, ^24^ During winter, the bacterial community was dominated by members of the phyla *Firmicutes, Bacteroidota* and *Proteobacteria*, which together correspond over 80% of the relative abundance. These bacterial phyla, specifically *Bacteroidota* and *Proteobacteria*, have also been reported in urban rivers worldwide with key roles in nutrient cycling and degradation of recalcitrant compounds.^25^, ^26^, ^27^, ^28^ At the family level, the most abundant taxa (> 5%) were *Lachnospiraceae* and *Ruminococcaceae* (*Firmicutes*), *Moraxellaceae* (*Gammaproteobacteria*), *Comamonadaceae* (*Betaproteobacteria*), *Arcobacteraceae* (*Campylobacterota*) and *Prevotellaceae* (*Bacteroidota*). Although *Moraxellaceae and Comamonadaceae* have been found in urban rivers in Europe,^25^^,^^31^ and Latin America^27^ and Asian^28^ these bacterial families identified herein have been previously proposed as indicators of faecal contamination, wastewater discharge and important carriers of antibiotic resistance. The bacterial families *Lachnospiraceae* and *Ruminococcaceae* are among the major taxa of the human gut microbiota^29^ and have also been detected in hospital and urban wastewater in the megacity of São Paulo.^15^ suggesting potential faecal contamination.^14^, ^15^, ^16^, ^17^, ^18^, ^19^, ^20^, ^21^, ^22^, ^23^, ^24^, ^25^, ^26^, ^27^, ^28^, ^29^, ^30^, ^31^ The families *Moraxellaceae* and *Comamonadaceae* have been shown to increase in abundance in rivers downstream of wastewater discharge.^31^^,^^32^ In addition, *Moraxellaceae* include clinically relevant pathogenic bacteria that exhibit broad spectrum of antibiotic resistance.^33^ Members of the *Arcobacteraceae* family include emerging antibiotic resistance enteropathogens such as *Arcobacter* spp., which has been abundantly found in the Pinheiros river that crossed São Paulo.^10^^,^^15^ This bacterial family have also been described in aquatic environments from Denmark, Australia and Korea.^34^ Finally, the *Prevotellaceae* family is primarily a gut-resident organism and its genus *Provatella* has been abundantly found in human faecal and sewage samples in Brazil.^14^ Statistically significant seasonal shifts (p < 0.05) were observed for *Ruminococcaceae, Arcobacteraceae, Flavobacteriaceae, Comamonadaceae, Aeromonadaceae* and *Rhodocyclaceae* families. In our study, temperature, precipitation and antimicrobials investigated seemed to not affect the bacterial population in the stream. The microbial variations observed might be due to other abiotic factors or, wastewater discharge from the nearby informal settlements, as already reported for *Ruminococcaceae, Comamonadaceae*, and *Rhodocyclaceae*.^31^ However, it is important to mention that in our study, we could not analyse a control (upstream or downstream) site, to properly assess the impact of the untreated effluent discharge into the urban stream. This limitation was due to the urban landscape of the city which has restricted access to most parts of the considered urban waterway.

To explore clinically relevant ARB, culture-dependent methods of two sampling campaigns were also realized. The bacterial strains recovered from the urban stream belong to the most abundant bacterial phyla found such as *Proteobacteria* and *Bacteroidota*. Overall*,* these isolates presented a high percentage of resistance mainly to third generation cephalosporins and carbapenems. These stream bacterial isolates harbour carbapenemases genes such as *bla*_VIM_ and *bla*_KPC_ detected by conventional PCR and, whole genome sequencing analysis performed reveals also a broad diversity of ARGs, efflux pumps and plasmids that might explain their resistance profile. Vancomycin-resistant enterococci along with ESBL ARGs were not found. Resistance to beta-lactam antibiotics was observed in all the *Enterobacteriaceae* bacterial isolates being the gene *bla*_TEM_ the most prevalent ESBL, present in 75% of the isolates (6 out of 8). Carbapenemase ARGs such as *bla*_KPC-2_, *bla*_GES-5,_ *bla*_OXA-1_ and AmpC types β-lactamases were also detected by WGS. Notably, the isolates *K. pneumoniae* SR5V4, *S. marcescens* SR6R19 and *Ent. cloacae* SR6V14 co-harbour ESBL and carbapenemase ARGs. Clinically relevant ESBL- and carbapenemase-producing *Enterobacteriaceae* have been detected in aquatic environments.^35^, ^36^, ^37^ In low- and middle-income countries, urban waterways are particularly vulnerable to ARB contamination due to anthropogenic pressures, especially the discharge of untreated domestic and industrial wastewater.^38^ Some stream *Enterobacteriaceae* displayed multidrug phenotypic resistance to aminoglycosides and quinolones and *Serratia marcescens* SR6-R19 isolate was resistant to all tested antibiotic classes and carried multiple ARGs. This species is considered an emerging, opportunistic hospital-acquired pathogen with high multidrug resistance potential.^38^ The bacterial isolates obtained also harbour a broad array of genes encoding antibiotic efflux pumps, likely reflecting their environmental origin.^39^ and potentially conferring multidrug-resistance profile. Furthermore, all the isolates were also characterized for their Chlorhexidine Minimum Inhibitory Concentration (CHX-MIC). The latter was determined because of its detection in the hospital and urban wastewater in São Paulo,^15^ together with the presence of colistin-resistant bacteria (Unpublished data) in the same water samples collected near the stream examined in this study. This is also supported by evidence from the literature suggesting that the presence of biocides may increase colistin resistance in carbapenem-resistant *Enterobacteriaceae*.^40^ Herein, we identified *Enterobacteriaceae* isolates such as *K. pneumoniae* and *S. marcescens* exhibiting high CHX-MIC values, most likely associated with the presence of genes such as *qac*Edelta1, *qac*L, and *qac*E, as well as the *Inc*HI2 plasmid.^41^^,^^42^

The genome sequencing of the *Enterobacteriaceae* isolates reveals the presence of several other *Inc*-type plasmids being the *Inc*FIB plasmid, the most common replicon type present in 62.5% of the isolates (5 out of 8). Plasmids belonging to the *Inc*F group are conjugative, mainly present in *Enterobacteriaceae* bacterial family, and responsible for the dissemination of ESBL genes and other determinants conferring resistance to aminoglycoside and quinolone antibiotics.^43^ Albeit we did not further investigate the backbone structure of the plasmids, the latter may facilitate the dissemination of critical ARGs in aquatic habitats among pathogenic bacteria.

Finally, the bacterial isolates possesses different encoding virulence factors, including toxin (*hly*A, *hly*E), adhesion (*fim*H, *yeh*A-D, *nlp*I), biofilm formation (*csg*A, *mrk*A), anti-complement factor (*tra*T), intestinal colonization (anr), in *E. coli, K. pneumoniae* and *C. amalonaticus* suggesting potential pathogenicity. Nonetheless, additional studies are needed to further characterize these isolates*.*

Under the *Gammaproteobacteria* family, the urban stream isolate *Aeromonas caviae* SR5V5 exhibited phenotypic resistance to β-lactams and aminoglycosides, likely due to the presence of *bla*_VIM_, *bla*_KPC_ and *acc*, respectively. Multidrug resistant *A. caviae* have recently been isolated from drinking water in Brazil^44^ as well as from effluent of watsterwater treatment plants in Japan^45^ and in aquatic food animals in Africa and Asia.^46^ The CHX-MIC values in *Aeromonas* spp. ranged between 8 and 32 mg/L, potentially associated with the presence of *qac* genes.

Concerning the presence of antimicrobial residues, levofloxacin and chlorhexidine were detected once in July and September, respectively. Azithromycin was consistently detected in all samples, whereas ceftriaxone was detected in August and September. In Brazil, similar concentrations values of these compounds in different water metrics have recently been described.^15^^,^^24^ Likewise, azithromycin has been detected in rivers in China^47^ and India,^48^ and in wastewater in Europe,^49^ while ceftriaxone has been detected in wastewater in India^50^ and Ethiopia.^51^ Predicted No Effect Concentrations (PNECs) for selection of resistance for these antibiotics have been established (0.25 and 0.032 μg/L for azithromycin and ceftriaxone, respectively)^52^ being our findings above of these values. However, despite the antibiotic compounds detected, a direct correlation with the presence of free ARGs or with the occurrence of ARB cannot be established due to the exploratory nature of this investigation and the limited number of samples analyzed. In addition, phenotypic resistance to azithromycin and its associated ARGs was not assessed, as antimicrobial susceptibility testing and molecular analyses focused on β-lactam resistance. Nonetheless, the simultaneous contamination of the urban stream by antimicrobial residues, free ARGs, and ARB should not be neglected, as it may represent a potential hotspot for the dissemination of antimicrobial resistance in the environment. Furthermore, the relatively small number of samples, influenced by resource constraints, represents an additional limitation of this study. Therefore, as this was an exploratory investigation, the findings should be interpreted as preliminary and warrant further investigation.

## Conclusion

Our study provides an overview of the bacterial community structure and AR contaminants (ARB, ARGs and antimicrobial residues) in the *Riacho Doce* stream, an urban waterway crossing an informal settlement in the megacity of São Paulo. Its microbial population includes taxa linked to fecal contamination, pathogenicity, and antibiotic resistance. These urban stream bacterial isolates presented resistance to clinically important beta-lactam antibiotics, including third-generation cephalosporin and carbapenems, a resistance recognized as a top-priority public health threat. WGS analysis reveals that strains co-harbour different arrays of ARGs, plasmids, efflux pumps and virulence genes. CHX-MIC testing revealed high MIC values for *K. pneumoniae* SR5V4 and *S. marcescens* SR6-R16 and SR6-R18. The urban stream was also contaminated by antibiotic residues such as azithromycin, ceftriaxone and levofloxacin. Their presence may indicate a potential risk, requiring further investigation and potential regulatory action. This underscores the urgent need for AR monitoring strategies in urban rivers as well as effective wastewater management to prevent urban rivers contamination and mitigate human and animal health risks.

## Data availability

All the data of this original research are present in the main Article text and in the Supplementary Material. The raw sequencing data of the 16S rRNA gene amplicon sequencing as well as of the WGS of the bacterial isolates have been submitted at the Sequence Read Archive (SRA) repository (https://www.ncbi.nlm.nih.gov/sra) under the corresponding BioProject accession number PRJNA1216305.

## Funding

This research did not receive any specific grant from funding agencies in the public, commercial, or not-for-profit sectors.

## Conflicts of interest

The authors declare that they have no known competing financial interests or personal relationships that could have appeared to influence the work reported in this paper.

## Appendix Supplementary materials

Image, application 1

Image, application 2
