# Supplementary material for: Insights of bacterial community structure and occurrence of antibiotic resistance and antimicrobial compounds in an urban stream in the megacity of São Paulo, Brazil
Source: Braz J Infect Dis. 2026 Apr 21;30(3):105818. doi: 10.1016/j.bjid.2026.105818 (PMC13121429; doi:10.1016/j.bjid.2026.105818)
Supplement: Supplementary file 1 [file mmc1.docx]

**BJID-D-25-00251_Supplementary Material**

**Table S1** Additional information about meteorological conditions, water samples filtered, DNA quantification and reads per sample.

| **Sample** | **Sampling date** | **Precipitation (mm)^a^** | **Ar temperature (°C)^b^** | **Water temperature (°C)** | **Amount of filtered water (mL)** | **DNA quantification (ng/μL)** | **Reads per sample** |
| --- | --- | --- | --- | --- | --- | --- | --- |
| SR3 | 28.06.21 | 0 | 14.6 | 20 | 150 | 23,5 | 86809 |
| SR4 | 12.07.21 | 0 | 12.7 | 19 | 100 | 6,74 | 46793 |
| SR5 | 26.07.21 | 0 | 12.9 | 19 | 150 | 13.9 | 65043 |
| SR6 | 09.08.21 | 0 | 13.3 | 19 | 150 | 10 | 98450 |
| SR7 | 23.08.21 | 0 | 16.6 | 18 | 120 | 12,7 | 147930 |
| SR8 | 09.09.21 | 3.1 | 20.5 | 19 | 120 | 11,3 | 148852 |
| SR9 | 20.09.21 | 0 | 18.8 | 19 | 100 | 13 | 80526 |

^a^ Precipitation data for the days of sampling were obtained by the CGE center (CGE, 2025).

^b^ Ar temperature data of the sampling days were downloaded by the INMET institute website (NMET, 2025). The values are the average of 3 temperatures measures (at 8, 9 and 10 AM when the samples were approx. collected).

**Table S2** Bacterial taxa (phylum and family) showing significant variations during the winter. The presented values are the average of the relative abundance of two samples of each month ± the standard deviation.

| **Bacterial taxa** | **July (SR4‒SR5)*** | **August (SR5‒SR6)** | **September (SR8‒SR9)** |
| --- | --- | --- | --- |
| *Firmicutes* | 34.9 ± 2.3^a^ | 31.5 ± 0.5^a,b^ | 28.9 ± 1.5^b^ |
| *Campilobacterota* | 4.4 ± 0.8^a^ | 7.8 ± 0.3^b^ | 9.6 ± 1.3^b^ |
| *Actinobacteriota* | 3.4 ± 0.3^a^ | 3.0 ± 0.1^a,b^ | 2.6 ± 0.1^b^ |
| *Ruminococcaceae* | 8.2 ± 0.2^a^ | 8.4 ± 0.0^a,b^ | 7.1 ± 0.2^c^ |
| *Veillonellaceae* | 1.5 ± 0.1^a^ | 1.4 ± 0.0^a,b^ | 1.1 ± 0.2^b^ |
| *Flavobacteriaceae* | 0.8 ± 0.1^a^ | 0.9 ± 0.0^a,b^ | 1.2 ± 0.1^b^ |
| *Comamonadaceae* | 4.3 ± 0.6^a^ | 4.0 ± 0.2^a^ | 5.3 ± 0.0^a,b^ |
| *Aeromonadaceae* | 0.7 ± 0.1^a^ | 1.7 ± 0.1^b^ | 1.2 ± 0.1^a,c^ |
| *Rhodocyclaceae* | 1.4 ± 0.0^a^ | 1.1 ± 0.0^a^ | 2.2 ± 0.6^b^ |
| *Arcobacteraceae* | 4.2 ± 0.7^a^ | 7.5 ± 0.3^b,c^ | 9.3 ± 1.4^c^ |

^a,b,c^ Statistically significant difference (p < 0.05) between months.

* The sample SR3 was excluded because it represented the only replicate of the month of June. For all the other months (July, August and September, the samples were collected twice.

**Table S3** Antibiotic Resistance Genes (ARGs), plasmids and virulence determinants of the sequenced urban stream bacterial isolates.

| **Bacterial isolates** | **Bacterial identification^a^** | **ARGs^b,c^** | **Antibiotic^d^** | **% Identity** | **Plasmids^e^** | **Virulence genes^f^** | **MLST^g^** |
| --- | --- | --- | --- | --- | --- | --- | --- |
| SR5R1 | *Escherichia coli* | *aph*(3')-Ia^*^; *aph*(3'')-Ib^*^; *aph*(6)-Id^*^; *aad*A5^*^; *aph*(3')-Ia^**^*; aph*(3'')-Ib^**^; *aph*(6)-Id^**^*; aad*A5^**^ | AG | 100; 100; 100; 100; 98; 99; 99; 100 | *Inc*Y; *Inc*Q1 | *csg*A*; fim*H*; ter*C*; yeh*; *asl*A; *fde*C; *hly*E; *iss; yehA; yehB; yehD* | Unknown |
|  |  | *bla*_TEM-1B_^*^; *bla*_TEM-1_^**^; *bla*_CTX-M-15_^*^; *bla*_CTX-M-15_^**^ | B | 100; 100; 100; 100 |  |  |  |
|  |  | *cat*A1^*^*; cat*A1^**^ | A | 100 |  |  |  |
|  |  | *dfr*A17 ^*^*; dfr*A17 ^**^ | DP | 99; 99 |  |  |  |
|  |  | *qac*E^*^; *qac*Edelta1^**^ | D | 100; 100 |  |  |  |
|  |  | *qnr*S1^*^ | Q | 99 |  |  |  |
|  |  | *sul*1^*^; *sul*2^*^ | S | 100; 99 |  |  |  |
|  |  | *tet*A^*^; *tet*B^*^; *tet*A^**^; *tet*R^**^ | T | 100; 99; 100; 100 |  |  |  |
|  |  | ***acr*D^**^; a*cr*E^**^; *acr*F^**^** | **MAR** | **100; 100; 100** |  |  |  |
|  |  | ***bac*A^**^** | **P** | **100; 100; 99** |  |  |  |
|  |  | ***bae*R^**^; *bae*S^**^** | **MAR** | **100; 100** |  |  |  |
|  |  | ***emr*A^**^; *emr*B^**^; *emr*K^**^; *emr*R^**^** | **Q; T** | **100; 99; 100; 99** |  |  |  |
|  |  | ***ept*A^**^** | **P** | **100** |  |  |  |
|  |  | ***evg*A^**^; *evg*S^**^** | **MAR** | **100; 100** |  |  |  |
|  |  | ***kdp*E^**^** | **A** | **100; 99** |  |  |  |
|  |  | ***mar*A^**^** | **MAR** | **100** |  |  |  |
|  |  | ***mdt*A^**^; *mdt*B^**^; mdtH^**^** | **AC; MAR** | **99, 99** |  |  |  |
|  |  | ***mdt*F^**^; *mdt*N^**^; *mdt*P^**^** | **MAR** | **100; 100; 100** |  |  |  |
|  |  | ***msb*A^**^** | **N** | **100** |  |  |  |
|  |  | ***pmr*F^**^** | **P** | **100** |  |  |  |
|  |  | ***tol*C^**^** | **MAR** | **100** |  |  |  |
|  |  | ***yoj*l^**^** | **P** | **99** |  |  |  |
| SR5R2 | *Escherichia coli* | *aph*(3')-Ia; *aph*(3'')-Ib; *aph(6)-Id; aph*(6)-Id*; aph*(3'')-Ib*; aph*(3')-Ia | AG | 100; 100; 100; 99; 99; 99 | *Inc*HI2; *Inc*HI2A; *Inc*FIB; *Inc*Q1 | *fim*H; *ter*C; *hly*A; *hly*E; *csg*A; *yeh*A; *yeh*B; *yeh*C; *fde*C; *yeh*D | 1201 |
|  |  | *bla*_CTX-M-2_^*^; bla_TEM-1B_^*^; bla_CTX-M-2_^**^; bla_EC-15_^**^ | B | 100; 100; 100; 98 |  |  |  |
|  |  | *dfr*A7^*^; *dfr*A7^**^; *dfr*A14^*^; | DP | 100; 99; 99 |  |  |  |
|  |  | *qac*E^*^; *qac*Edelta1^**^ | D | 100; 100 |  |  |  |
|  |  | *qnrS1^*^; qnrS1^**^* | Q | 100; 100 |  |  |  |
|  |  | *sul*1^*^; *sul*2^*^; *sul*1^**^; *sul*2^**^ | S | 100; 100; 100; 99 |  |  |  |
|  |  | *tet*A^*^; *tet*A^**^ | T | 100; 99 |  |  |  |
|  |  | ***acrD^**^; acrF^**^; acrB^**^*** | **AG; MAR** | **99; 99; 99** |  |  |  |
|  |  | ***acr*E^**^; a*cr*S^**^** | **MAR** | **100** |  |  |  |
|  |  | ***bac*A^**^** | **P** | **100** |  |  |  |
|  |  | ***bae*S^**^; *bae*R^**^** | **MAR** | **99; 99** |  |  |  |
|  |  | ***cpx*A^**^** | **MAR** | **100** |  |  |  |
|  |  | ***emr*A^**^; *emr*B^**^; *emr*R^**^; *emr*K^**^; *emr*Y^**^** | **Q; T** | **100; 100; 100; 100; 99; 99** |  |  |  |
|  |  | ***evg*A^**^*; evg*S^**^** | **MAR** | **100; 99** |  |  |  |
|  |  | ***gad*X^**^** | **MAR** | **98** |  |  |  |
|  |  | ***kdpE*^**^** | **AG** | **10000%** |  |  |  |
|  |  | ***mar*A^**^** | **MAR** | **100** |  |  |  |
|  |  | ***mdt*B^**^; *mdt*C^**^; *mdt*E^**^; *mdt*F^**^; *mdt*G^**^; *mdt*H^**^; *mdt*M^**^; *mdt*N^**^; *mdt*O^**^; *mdt*P^**^** | **AC; MAR** | **100; 99; 97; 99; 100; 100; 99; 99; 99; 98** |  |  |  |
|  |  | ***msb*A^**^** | **N** | **100** |  |  |  |
|  |  | ***pmr*F^**^** | **P** | **100** |  |  |  |
|  |  | ***tol*C^**^** | **MAR** | **100** |  |  |  |
|  |  | **y*oj*I^**^** | **P** | **99** |  |  |  |
| SR5V4 | *Klebsiella pneumoniae* | *aac*(6')-Ib3^*^*; aad*A1^**^ | AG | 100; 99 | *Col*(pHAD2); *Col*440I; *Inc*FII; *Inc*FIB; *Inc*R | *fim*H; *tra*T; *iut*A; *mrk*A; *nlpI* | 2144 |
|  |  | *arn*T^**^; *ept*B^**^ | P | 97; 98 |  |  |  |
|  |  | *bla*_KPC-2_^*^; *bla*_KPC-2_^**^; *bla*_GES-5_^*^; *bla*_TEM-15_^**^; *bla*_TEM-52_^*^; *bla*_TEM-197_^*^; *bla*_OKP-B-1_ | B | 100; 100; 100; 100; 99; 99; 99 |  |  |  |
|  |  | *fos*A5^*^; *fos*A6^**^ | F | 96; 97 |  |  |  |
|  |  | ***omp*A** | **MAR** | **99** |  |  |  |
|  |  | ***qac*L^*^; *qac*E^*^; *qac*L^**^; *qac*Edelta1^**^** | **D** | **99; 100; 98; 100** |  |  |  |
|  |  | ***sat*2** | **AG** | **100** |  |  |  |
|  |  | ***sul1*^*^; *sul1^**^*** | **S** | **100: 100** |  |  |  |
|  |  | ***bae*R^**^** | **MAR** | **92** |  |  |  |
|  |  | ***crp*^**^** | **MAR** | **99** |  |  |  |
|  |  | ***emr*R^**^** | **Q** | **93** |  |  |  |
|  |  | ***hns^**^*** | **MAR** | **94** |  |  |  |
|  |  | ***lpt*D^**^** | **MAR** | **99** |  |  |  |
|  |  | ***mar*A^**^** | **MAR** | **99** |  |  |  |
|  |  | ***msb*A^**^** | **N** | **93** |  |  |  |
|  |  | ***oqx*A^**^; o*qx*B^**^** | **Q** | **97; 95** |  |  |  |
| SR5V5 | *Aeromonas caviae* | *aac*(6')-Ib-cr^*^; *aac*(3)-Iid^*^; *aac*(3)-Iid^**^; *aac*(6')-Ib-cr6^**^ | AG | 100; 99; 100; 99 | *Inc*P6*; Inc*Q2 | *clpK*2 | Unknown |
|  |  | *arr*-3^*^; *arr*-3^**^ | R | 100; 100 |  |  |  |
|  |  | *bla*_KPC-2_^*^; bla_KPC-2_^**^; bla_TLA-1_^*^; bla_MOX-6_^*^; bla_TEM-1C_^*^; bla_TEM-1A_^*^; bla_TEM-40_^*^; bla_TEM-150_^*^; bla_TEM-171_^*^; bla_OXA-504_^**^ | B | 100; 100; 94; 97; 99; 99; 99; 99; 99; 98 |  |  |  |
|  |  | *mph*A^*^; *mph*E^*^; *mph*A^**^; *mph*E^**^ | M | 100; 99; 100; 99 |  |  |  |
|  |  | *mrx^**^* | M | 99 |  |  |  |
|  |  | *qac*E^*^; *qac*Edelta1^**^ | D | 100; 100 |  |  |  |
|  |  | *sul*1^*^ | S | 99 |  |  |  |
|  |  | *tet*C^*^; *tet*C^**^ | T | 99; 100 |  |  |  |
|  |  | ***msr*E^**^** | **MAR** | **100** |  |  |  |
| SR6R16 | *Serratia marcescens* subsp. marcescens Db11 | *aac*(6')-Ib-cr^*^; *aac*(6')-Ic^*^; *aph*(3')-Via^*^; *acc*(6')-Ic^**^; *acc*(6')-Ic^**^*; acc*(6')-Ib-cr6^**^ | AG | 100; 96; 98; 97; 98; 99 | *Inc*HI2*; Inc*HI2A; *Inc*Q1; *Inc*P6; *Inc*FIB(K); *Col*440II; | *ter*C | Unknown |
|  |  | *bla*_OXA-1_^*^; *bla*_SRT-2_^*^; *bla*_OXA-1_^**^; *bla*_SRT-2_^**^ | B | 100; 97; 100; 99 |  |  |  |
|  |  | *cat*B3^*^ | A | 100 |  |  |  |
|  |  | *dfr*A14^*^; *dfr*A14^**^ | DP | 100; 99 |  |  |  |
|  |  | ***crp***** | **MAR** | **99** |  |  |  |
| SR6R18 | *Serratia marcescens* subsp. marcescens Db11 | *aac*(6')-Ib-cr^*^; *aph*(3')-Via^*^; *acc*(6')-Ib-cr6^**^; *aph*(3')-Via^**^ | AG | 100; 98; 99; 98 | *Inc*HI2; *Inc*HI2A; *Inc*Q1; *Inc*P6; *Inc*FIB(K); *Col*440I; *Col*440II | *ter*C | Unknown |
|  |  | *bla*_KPC-2_^*^; *bla*_OXA-1_^*^; *bla*_KPC-2_^**^; *bla*_OXA-1_^**^*; bla*_TEM-1C_^*^; *bla*_TEM-1A_^*^; *bla*_TEM-40_^*^; *bla*_TEM-150_^*^; *bla*_TEM-171_^*^ | B | 100; 100; 100; 100; 100; 100; 100; 100; 100 |  |  |  |
|  |  | *cat*B3^*^ | A | 100 |  |  |  |
|  |  | *dfr*A14^*^; *dfr*A14^**^ | DP | 100; 100 |  |  |  |
|  |  | ***crp^**^*** | **MAR** | **99** |  |  |  |
| SR6R19 | *Serratia marcescens* subsp. marcescens Db11 | *aac*(6')-Ib-cr^*^; *aac*(6')-Ic^*^; *aph*(6)-Id^*^; *aac*(3)-Iia^*^; *aph*(3'')-Ib^*^; *aad*A1^*^; *aph*(3'')-Ib^**^; *aac*(6')-Ic^**^; *aac*(3)-Iie^**^; *aac*(6')-Ib-cr6^**^; *aad*A1^**^ | AG | 100; 96; 100; 100; 100; 100; 99; 99; 99; 99; 99 | *Inc*HI2; *Inc*HI2A | *terC* | Unknown |
|  |  | *bla*_OXA-1_^*^; *bla*_TEM-1B_^*^; *bla*_CTX-M-15_^*^; *bla*_SRT-2_^*^; *bla*_OXA-1_^**^; *bla*_TEM-1B_^**^ | B | 100; 100; 100; 97; 100; 100 |  |  |  |
|  |  | *cat*B3^*^ | A | 100 |  |  |  |
|  |  | *qnr*B1^*^ | Q | 99 |  |  |  |
|  |  | *sul*2^*^; *sul*2^**^ | S | 100; 100 |  |  |  |
|  |  | *tet*A^*^ | T | 95 |  |  |  |
|  |  | ***crp*^**^** | **MAR** | **99** |  |  |  |
| SR6V14 | *Enterobacter cloacae* | *aac*(6')-Ib-cr^*^; *aac*(6')-Ib3^*^; *aac*(6')-Ib3^**^ | AG | 100; 100; 100 | pKPC-CAV1321; *Inc*P1; *Inc*P6; *Inc*X3; *Inc*X5 | *nlpI* | Unknown |
|  |  | *bla*_TEM-1C_^*^; *bla*_TEM-1A_^*^; *bla*_TEM-40_^*^; *bla*_TEM-150_^*^; *bla*_TEM-171_^*^; *bla*_ACT-9_^*^; *bla*_ACT-28_^**^; | B | 100; 100; 100; 100; 100; 100; 99 |  |  |  |
|  |  | *dfr*A1^*^; dfrA1^**^ | DP | 100; 100 |  |  |  |
|  |  | *qac*L^*^, *qac*E^*^; *qac*Edelta1^**^, *qac*L^**^ | D | 99; 100; 100; 98 |  |  |  |
|  |  | *rmt*G^*^; *rmt*G^**^ | AG | 100; 100 |  |  |  |
|  |  | *sul*1^*^ | S | 100 |  |  |  |
| SR6V15 | *Chryseobacterium flavum* | NF | ‒ | ‒ | NF | NF | Unknown |
| SR6V16 | *Chryseobacterium gambrini* | NF | ‒ | ‒ | NF | NF | Unknown |
| SR6V17 | *Chryseobacterium hispalense* | NF | ‒ | ‒ | NF | NF | Unknown |
| SR6M19 | *Chryseobacterium hispalense* | NF | ‒ | ‒ | NF | NF | Unknown |
| SR6M1 | *Citrobacter amalonaticus* | *bla*_KPC-2_^*^; bla_KPC-2_^**^ | B | 100; 100 | *Inc*FIB(K); *Inc*FII(Yp); *Inc*X3; *Col*440I | *anr*; *nlpI; clpK*2; *mrk*A; *tra*T | Unknown |
|  |  | *mph*A^*^; *mph*E^*^; *mph*A^**^ | M | 100; 99; 100 |  |  |  |
|  |  | *msr*E^*^; msrE^**^ | MAR | 100; 100 |  |  |  |
|  |  | *qac*E^*^; *qac*Edelta1^**^ | D | 100; 100 |  |  |  |
|  |  | *qnr*VC4^*^; *qnr*VC4^**^ | Q | 100; 100 |  |  |  |
|  |  | *sul*1^*^; *sul*1^**^ | S | 100; 100 |  |  |  |
|  |  | ***acr*B^**^** | **MAR** | **95** |  |  |  |
|  |  | ***bac*A^**^** | **P** | **97** |  |  |  |
|  |  | ***bae*R^**^** | **MAR** | **96** |  |  |  |
|  |  | ***cml*A1^**^; *cml*A5^**^** | **MAR** | **99; 100** |  |  |  |
|  |  | ***crp*^**^** | **MAR** | **99** |  |  |  |
|  |  | ***emr*R^**^** | **Q** | **92** |  |  |  |
|  |  | ***hns*^**^** | **MAR** | **96** |  |  |  |
|  |  | ***mar*A^**^** | **MAR** | **96** |  |  |  |
|  |  | ***mdt*B^**^** | **AC; MAR** | **94** |  |  |  |
|  |  | ***msb*A^**^** | **N** | **97** |  |  |  |
| SR6M23 | *Comamonas jiangduensis* | NF | ‒ | ‒ | NF | NF | Unknown |
| SR6M24 | *Comamonas jiangduensis* | *aad*A1^*^; *aad*A1^**^ | AG | 100; 99 | NF | NF | Unknown |
|  |  | *cat*B3^*^; *cat*B3^**^ | A | 100; 100 |  |  |  |
|  |  | *dfr*A21^*^; dfrA21^**^ | DP | 100; 100 |  |  |  |
|  |  | *mph*E^*^; *mph*E^**^ | M | 100; 100 |  |  |  |
|  |  | *qac*E^*^; *qac*Edelta1^**^ | D | 100; 100 |  |  |  |
|  |  | *sul*1*^*^; sul*1^**^ | S | 100; 100 |  |  |  |
|  |  | ***msr*E*^*^; msr*E^**^** | MAR | 100; 100 |  |  |  |
| SR6M25 | *Comamonas jiangduensis* | NF | ‒ | ‒ | NF | NF | Unknown |

^a^ Bacterial species were confirmed using the SpeciesFinder-2.0 tool (https://cge.food.dtu.dk/services/SpeciesFinder/).

^b^ Antibiotic resistance genes (ARGs) were seek using the following database: ResFinder-4.5.0 (http://genepi.food.dtu.dk/resfinder)* and RGI (Resistance Gene Identifier) from CARD (Comprehensive Antibiotic Resistance Database) platform (https://card.mcmaster.ca/home)**.

^c^ In bold, efflux-pump-mediated resistance genes.

^d^ Antibiotic and other compounds: A, Amphenicols; AC, Aminocoumarins; AG, Aminoglycosides; B, Beta-lactams; D, Disinfectants and antiseptics; DP, Diaminopyrimidines; F, Fosfomicin; M, Macrolides; MAR, Multiple Antibiotic Resistance; N, Nitroimidazoles; P, Polypeptides; Q, Quinolones; R, Rifamycins; S, Sulfonamides; T, Tetracyclines.

^e^ Plasmids were found using the PlasmidFinder-2.0 too (https://cge.food.dtu.dk/services/PlasmidFinder/).

^f^ Virulance genes were screed using the database: VirulenceFinder-2.0 (https://cge.food.dtu.dk/services/VirulenceFinder/).

^g^ The multilocus sequence typing were infered by the tools MLST-2.0 tool (https://cge.food.dtu.dk/services/MLST/) and PubMLST (https://pubmlst.org/).

The threshold values used for all databases was > 90% similarity. NF, Not Found.

**Figure S1** Relative abundance of family’s taxa of water samples collected from the urban stream “*Riacho Doce*” from June to September 2021 (winter season). Only major taxa (> 5% total abundance) were included in the graph. Families with abundances < 5% are designated as “Others”.
