# Supplementary material for: Insights of bacterial community structure and occurrence of antibiotic resistance and antimicrobial compounds in an urban stream in the megacity of São Paulo, Brazil
Source: Braz J Infect Dis. 2026 Apr 21;30(3):105818. doi: 10.1016/j.bjid.2026.105818 (PMC13121429; doi:10.1016/j.bjid.2026.105818)
Supplement: Supplementary file 2 [file mmc2.pdf]

Supplementary Information

Research paper title:

Exploring antibiotic resistance, pharmaceutical compounds and bacterial community structure in an urban stream in the municipality of São Paulo, Brazil.

Authors:

Nazareno Scaccia[1,2,3], Maria Tereza Pepe Razzolini[4], Gabrielly Lacerda de Aragão[1,2], Joyce Vanessa da Silva Fonseca[1], Nilo José Coelho Duarte[5], Léonard de Vinci Kanda Kupa[5], Jonathan Cawettiere Espíndola[6], Ester Cerdeira Sabino[2,7], Anna Sara Levin[1,2,7], Silvia Figueiredo Costa[1,2].

1, Centres for Antimicrobial Optimisation Network Brazil, Departamento de Infectologia e Medicina Tropical, Faculdade de Medicina FMUSP, Universidade de Sao Paulo, Av. Dr. Enéas de Carvalho Aguiar, 470, 05403-000 Cerqueira César, Sao Paulo, SP, BR. **2**, Departamento de Infectologia e Medicina Tropical, Faculdade de Medicina FMUSP, Universidade de Sao Paulo, Av. Dr. Enéas de Carvalho Aguiar, 470, 05403-000 Cerqueira César, Sao Paulo, SP, BR. **3**, Departamento de Patologia, Centro Universitário Faculdade de Medicina do ABC (FMABC), Av. Príncipe de Gales, 821, 09060-650, Vila Príncipe de Gales, Santo André, SP, BR. **4**, Departamento de Saúde Ambiental, Faculdade de Saúde Pública FSP, Universidade de Sao Paulo, Av. Dr. Arnaldo, 715, 01246-904 Cerqueira César, Sao Paulo, SP, BR. **5**, Divisão de Laboratório Central, Hospital das Clinicas HCFMUSP, Faculdade de Medicina, Universidade de Sao Paulo, Av. Dr. Enéas Carvalho de Aguiar, 155, 01246-100 Cerqueira César, Sao Paulo, SP, BR. **6**, Escola Politécnica, Departamento de Engenheria Hidráulica e Ambiental, Centro Internacional de Referência em Reúso de Água CIRRA, Universidade de Sao Paulo, Cidade Universitária, 05508-020 Butantã, Sao Paulo, SP, BR. **7**, Hospital das Clinicas HCFMUSP, Faculdade de Medicina, Universidade de Sao Paulo, Av. Dr. Enéas Carvalho de Aguiar, 155, 01246-100 Cerqueira César, Sao Paulo, SP, BR.

Correspondence:

Nazareno Scaccia, PhD

Departamento de Infectologia e Medicina Tropical,

Faculdade de Medicina FMUSP, Universidade de Sao Paulo,

Avenida Dr. Enéas de Carvalho Aguiar, 470

05403-000, Cerqueira César, Sao Paulo, SP, BR.

E-mail: nazareno.scaccia@usp.br

Table S1. Additional information about meteorological conditions, water samples filtered, DNA quantification and reads per sample.

Sample	Sampling date	Precipitation (mm)[1]	Ar temperature (°C)[2]	Water temperature (ºC)	Amount of filtered water (ml)	DNA quantification (ng/µL)	Reads per sample
SR3	28.06.21	0	14.6	20	150	23,5	86809
SR4	12.07.21	0	12.7	19	100	6,74	46793
SR5	26.07.21	0	12.9	19	150	13.9	65043
SR6	09.08.21	0	13.3	19	150	10	98450
SR7	23.08.21	0	16.6	18	120	12,7	147930
SR8	09.09.21	3.1	20.5	19	120	11,3	148852
SR9	20.09.21	0	18.8	19	100	13	80526

1, precipitation data for the days of sampling were obtained by the CGE center (CGE, 2025).

2, Ar temperature data of the sampling days were downloaded by the INMET institute website (NMET, 2025). The values are the average of 3 temperatures measures (at 8, 9 and 10 AM when the samples were approx. collected).

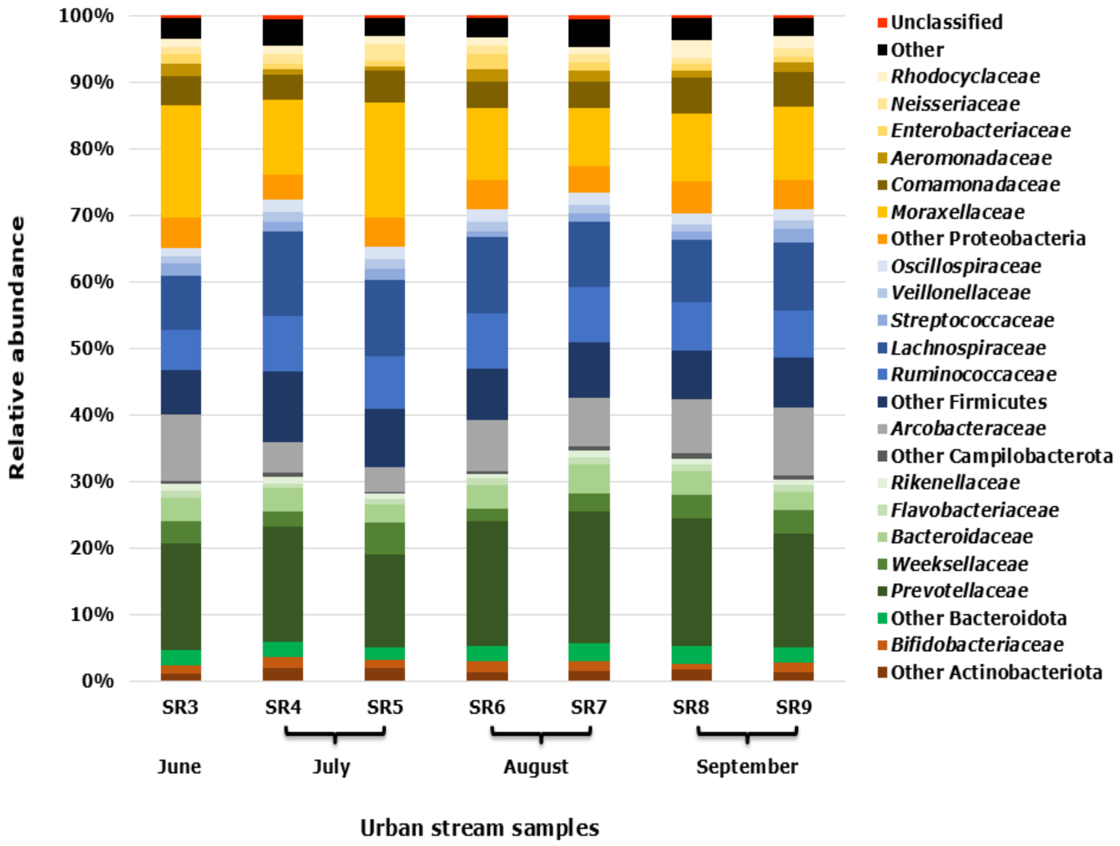

Figure S1. Relative abundance of families taxa of water samples collected from the urban stream "*Riacho Doce*" from June to September 2021 (winter season). Only major taxa (>5% total abundance) were included in the graph. Families with abundances <5% are designated as "Others".

Table S2. Bacterial taxa (phylum and family) showing significant variations during the winter. The presented values are the average of the relative abundance of two samples of each month ± the standard deviation.

Bacterial taxa	July (SR4-SR5)*	August (SR5-SR6)	September (SR8-SR9)
Firmicutes	34.9 ± 2.3 a	31.5 ± 0.5 a,b	28.9 ± 1.5 b
Campilobacterota	4.4 ± 0.8 a	7.8 ± 0.3 b	9.6 ± 1.3 b
Actinobacteriota	3.4 ± 0.3 a	3.0 ± 0.1 a,b	2.6 ± 0.1 b
Ruminococcaceae	8.2 ± 0.2 a	8.4 ± 0.0 a,b	7.1 ± 0.2 c
Veillonellaceae	1.5 ± 0.1 a	1.4 ± 0.0 a,b	1.1 ± 0.2 b
Flavobacteriaceae	0.8 ± 0.1 a	0.9 ± 0.0 a,b	1.2 ± 0.1 b
Comamonadaceae	4.3 ± 0.6 a	4.0 ± 0.2 a	5.3 ± 0.0 a,b
Aeromonadaceae	0.7 ± 0.1 a	1.7 ± 0.1 b	1.2 ± 0.1 a,c
Rhodocyclaceae	1.4 ± 0.0 a	1.1 ± 0.0 a	2.2 ± 0.6 b
Arcobacteraceae	4.2 ± 0.7 a	7.5 ± 0.3 b,c	9.3 ± 1.4 c

Legend: a-c; statistically significant difference ($P < 0.05$) between months.

*, the sample SR3 was excluded because it represented the only replicate of the month of June. For all the other months (July, August and September, the samples were collected twice.

Table S3. Antibiotic resistance genes (ARGs), plasmids and virulence determinants of the sequenced urban stream bacterial isolates.

Bacterial isolates	Bacterial identification[a]	ARGs[b,c]	Antibiotic[d]	% Identity	Plasmids[e]	Virulence genes[f]	MLST[g]
SR5R1	Escherichia coli	aph(3')-Ia*; aph(3")-Ib*; aph(6)-Id*; aadA5*; aph(3')-Ia**; aph(3")-Ib**; aph(6)-Id**; aadA5**	AG	100; 100; 100; 100; 98; 99; 99; 100	IncY; IncQ1	csgA; fimH; terC; yeh; aslA; fdeC; hlyE; iss; yehA; yehB; yehD	Unknown
		bla_{TEM-1B}*; bla_{TEM-1}**; $bla_{CTX-M-15}$*; $bla_{CTX-M-15}$**	B	100; 100; 100; 100			
		catA1*; catA1**	A	100			
		dfrA17 *; dfrA17 **	DP	99; 99			
		qacE*; qacEdelta1**	D	100; 100			
		qnrS1*	Q	99			
		sul1*; sul2*	S	100; 99			
		tetA*; tetB*; tetA**; tetR**	T	100; 99; 100; 100			
		__acrD**; acrE**; acrF**__	__MAR__	__100; 100; 100__			
		__bacA**__	__P__	__100; 100; 99__			
		__baeR**; baeS**__	__MAR__	__100; 100__			
		__emrA**; emrB**; emrK**; emrR**__	__Q; T__	__100; 99; 100; 99__			
		__eptA**__	__P__	__100__			
		__evgA**; evgS**__	__MAR__	__100; 100__			
		__kdpE**__	__A__	__100; 99__			
		__marA**__	__MAR__	__100__			
		__mdtA**; mdtB**; mdtH**__	__AC; MAR__	__99, 99__			
		__mdtF**; mdtN**; mdtP**__	__MAR__	__100; 100; 100__			
		__msbA**__	__N__	__100__			
		__pmrF**__	__P__	__100__			
		__tolC**__	__MAR__	__100__			
		__yojI**__	__P__	__99__			
SR5R2	Escherichia coli	aph(3')-Ia; aph(3")-Ib; aph(6)-Id; aph(6)-Id; aph(3")-Ib; aph(3')-Ia	AG	100; 100; 100; 99; 99; 99	IncHI2; IncHI2A; IncFIB; IncQ1	fimH; terC; hlyA; hlyE; csgA; yehA; yehB; yehC; fdeC; yehD	1201
		$bla_{CTX-M-2}$*; bla_{TEM-1B}*; $bla_{CTX-M-2}$**; bla_{EC-15}**	B	100; 100; 100; 98			
		dfrA7*; dfrA7**; dfrA14*;	DP	100; 99; 99			
		qacE*; qacEdelta1**	D	100; 100			
		qnrS1*; qnrS1**	Q	100; 100			
		sul1*; sul2*; sul1**; sul2**	S	100; 100; 100; 99			
		tetA*; tetA**	T	100; 99			
		__acrD**; acrF**; acrB**__	__AG; MAR__	__99; 99; 99__			
		__acrE**; acrS**__	__MAR__	__100__			
		__bacA**__	__P__	__100__			
		__baeS**; baeR**__	__MAR__	__99; 99__			

		_cpx_A**<br>_emr_A**; _emr_B**; _emr_R**;<br>_emr_K**; _emr_Y**<br>_evg_A**; _evg_S**<br>_gad_X**<br>_kdpE_**<br>_mar_A**<br>_mdt_B**; _mdt_C**; _mdt_E**;<br>_mdt_F**; _mdt_G**; _mdt_H**;<br>_mdt_M**; _mdt_N**; _mdt_O**;<br>_mdt_P**<br>_msb_A**<br>_pmr_F**<br>_tol_C**<br>_yoj_I**	MAR<br>Q; T<br><br>MAR<br>MAR<br>AG<br>MAR<br>AC; MAR<br><br><br><br>N<br>P<br>MAR<br>P	100<br>100; 100; 100;<br>100; 99; 99<br>100; 99<br>98<br>10000%<br>100<br>100; 99; 97; 99;<br>100; 100; 99; 99;<br>99; 98<br><br>100<br>100<br>100<br>99			
SR5V4	_Klebsiella pneumoniae_	_aac_(6')-Ib3*; _aad_A1**<br>_arn_T**; _ept_B**<br>_bla_$_{KPC-2}$*; _bla_$_{KPC-2}$**; _bla_$_{GES-5}$*;<br>_bla_$_{TEM-15}$**; _bla_$_{TEM-52}$*; _bla_$_{TEM-197}$*; _bla_$_{OKP-B-1}$<br>_fos_A5*; _fos_A6**<br>_omp_A<br>_qac_L*; _qac_E*; _qac_L**;<br>_qac_Edelta1**<br>_sat_2<br>_sul1_*; _sul1_**<br>_bae_R**<br>_crp_**<br>_emr_R**<br>_hns_**<br>_lpt_D**<br>_mar_A**<br>_msb_A**<br>_oqx_A**; _oqx_B**	AG<br>P<br>B<br><br>F<br>MAR<br>D<br><br>AG<br>S<br>MAR<br>MAR<br>Q<br>MAR<br>MAR<br>MAR<br>N<br>Q	100; 99<br>97; 98<br>100; 100; 100;<br>100; 99; 99; 99<br>96; 97<br>99<br>99; 100; 98; 100<br><br>100<br>100: 100<br>92<br>99<br>93<br>94<br>99<br>99<br>93<br>97; 95	_Col_(pHAD2);<br>_Col_440I; _Inc_FII;<br>_Inc_FIB; _Inc_R	_fim_H; _tra_T; _iut_A;<br>_mrk_A;<br>_nlpI_	2144
SR5V5	_Aeromonas caviae_	_aac_(6')-Ib-cr*; _aac_(3)-Iid*;<br>_aac_(3)-Iid**; _aac_(6')-Ib-cr6**<br>_arr_-3*; _arr_-3**<br>_bla_$_{KPC-2}$*; bla$_{KPC-2}$**; bla$_{TLA-1}$*;<br>bla$_{MOX-6}$*; bla$_{TEM-1C}$*; bla$_{TEM-1A}$*;<br>bla$_{TEM-40}$*; bla$_{TEM-150}$*; bla$_{TEM-171}$*; bla$_{OXA-504}$**<br>_mph_A*; _mph_E*; _mph_A**; _mph_E**<br>_mrx_**<br>_qac_E*; _qac_Edelta1**	AG<br><br>R<br>B<br><br><br>M<br>M<br>D	100; 99; 100; 99<br><br>100; 100<br>100; 100; 94; 97;<br>99; 99; 99; 99;<br>99; 98<br>100; 99; 100; 99<br>99<br>100; 100	_Inc_P6; _Inc_Q2	_clpK_2	Unknown

		*sul*1*<br>*tet*C*; *tet*C**<br>***msr*E****	S<br>T<br>**MAR**	99<br>99; 100<br>**100**			
SR6R16	*Serratia marcescens* subsp. marcescens Db11	*aac*(6')-Ib-cr*; *aac*(6')-Ic*; *aph*(3')-Via*; *acc*(6')-Ic**; *acc*(6')-Ic**; *acc*(6')-Ib-cr6**<br>*bla*$_{OXA-1}$*; *bla*$_{SRT-2}$*; *bla*$_{OXA-1}$**; *bla*$_{SRT-2}$**<br>*cat*B3*<br>*dfr*A14*; *dfr*A14**<br>***crp*****	AG<br>B<br>A<br>DP<br>**MAR**	100; 96; 98; 97; 98; 99<br>100; 97; 100; 99<br>100<br>100; 99<br>**99**	*Inc*HI2; *Inc*HI2A; *Inc*Q1; *Inc*P6; *Inc*FIB(K); *Col*440II;	*ter*C	Unknown
SR6R18	*Serratia marcescens* subsp. marcescens Db11	*aac*(6')-Ib-cr*; *aph*(3')-Via*; *acc*(6')-Ib-cr6**; *aph*(3')-Via**<br>*bla*$_{KPC-2}$*; *bla*$_{OXA-1}$*; *bla*$_{KPC-2}$**; *bla*$_{OXA-1}$**; *bla*$_{TEM-1C}$*; *bla*$_{TEM-1A}$*; *bla*$_{TEM-40}$*; *bla*$_{TEM-150}$*; *bla*$_{TEM-171}$*<br>*cat*B3*<br>*dfr*A14*; *dfr*A14**<br>***crp*****	AG<br>B<br>A<br>DP<br>**MAR**	100; 98; 99; 98<br>100; 100; 100; 100; 100; 100; 100; 100; 100<br>100<br>100; 100<br>**99**	*Inc*HI2; *Inc*HI2A; *Inc*Q1; *Inc*P6; *Inc*FIB(K); *Col*440I; *Col*440II	*ter*C	Unknown
SR6R19	*Serratia marcescens* subsp. marcescens Db11	*aac*(6')-Ib-cr*; *aac*(6')-Ic*; *aph*(6)-Id*; *aac*(3)-Iia*; *aph*(3")-Ib*; *aad*A1*; *aph*(3")-Ib**; *aac*(6')-Ic**; *aac*(3)-Iie**; *aac*(6')-Ib-cr6**; *aad*A1**<br>*bla*$_{OXA-1}$*; *bla*$_{TEM-1B}$*; *bla*$_{CTX-M-15}$*; *bla*$_{SRT-2}$*; *bla*$_{OXA-1}$**; *bla*$_{TEM-1B}$**<br>*cat*B3*<br>*qnr*B1*<br>*sul*2*; *sul*2**<br>*tet*A*<br>***crp*****	AG<br>B<br>A<br>Q<br>S<br>T<br>**MAR**	100; 96; 100; 100; 100; 100; 99; 99; 99; 99; 99<br>100; 100; 100; 97; 100; 100<br>100<br>99<br>100; 100<br>95<br>**99**	*Inc*HI2; *Inc*HI2A	*terC*	Unknown
SR6V14	*Enterobacter cloacae*	*aac*(6')-Ib-cr*; *aac*(6')-Ib3*; *aac*(6')-Ib3**<br>*bla*$_{TEM-1C}$*; *bla*$_{TEM-1A}$*; *bla*$_{TEM-40}$*; *bla*$_{TEM-150}$*; *bla*$_{TEM-171}$*; *bla*$_{ACT-9}$*; *bla*$_{ACT-28}$**;<br>*dfr*A1*; dfrA1**<br>*qac*L*, *qac*E*; *qac*Edelta1**, *qac*L**<br>*rmt*G*; *rmt*G**<br>*sul*1*	AG<br>B<br>DP<br>D<br>AG<br>S	100; 100; 100<br>100; 100; 100; 100; 100; 100; 99<br>100; 100<br>99; 100; 100; 98<br>100; 100<br>100	pKPC-CAV1321; *Inc*P1; *Inc*P6; *Inc*X3; *Inc*X5	*nlpI*	Unknown

SR6V15	*Chryseobacterium flavum*	NF	-	-	NF	NF	Unknown
SR6V16	*Chryseobacterium gambrini*	NF	-	-	NF	NF	Unknown
SR6V17	*Chryseobacterium hispalense*	NF	-	-	NF	NF	Unknown
SR6M19	*Chryseobacterium hispalense*	NF	-	-	NF	NF	Unknown
SR6M1	*Citrobacter amalonaticus*	*bla*$_{KPC-2}$*; bla$_{KPC-2}$**<br>*mph*A*; *mph*E*; *mph*A**<br>*msr*E*; msrE**<br>*qac*E*; *qac*Edelta1**<br>*qnr*VC4*; *qnr*VC4**<br>*sul*1*; *sul*1**<br>***acr*B****<br>***bac*A****<br>***bae*R****<br>***cml*A1**; *cml*A5****<br>***crp*****<br>***emr*R****<br>***hns*****<br>***mar*A****<br>***mdt*B****<br>***msb*A****	B<br>M<br>MAR<br>D<br>Q<br>S<br>**MAR**<br>**P**<br>**MAR**<br>**MAR**<br>**MAR**<br>**Q**<br>**MAR**<br>**MAR**<br>**AC; MAR**<br>**N**	100; 100<br>100; 99; 100<br>100; 100<br>100; 100<br>100; 100<br>100; 100<br>**95**<br>**97**<br>**96**<br>**99; 100**<br>**99**<br>**92**<br>**96**<br>**96**<br>**94**<br>**97**	*Inc*FIB(K); *Inc*FII(Yp); *Inc*X3; *Col*440I	*anr*; *nlpI; clpK*2; *mrk*A; *tra*T	Unknown
SR6M23	*Comamonas jiangduensis*	NF	-	-	NF	NF	Unknown
SR6M24	*Comamonas jiangduensis*	*aad*A1*; *aad*A1**<br>*cat*B3*; *cat*B3**<br>*dfr*A21*; dfrA21**<br>*mph*E*; *mph*E**<br>*qac*E*; *qac*Edelta1**<br>*sul*1*; *sul*1**<br>***msr*E*; msrE****	AG<br>A<br>DP<br>M<br>D<br>S<br>MAR	100; 99<br>100; 100<br>100; 100<br>100; 100<br>100; 100<br>100; 100<br>100; 100	NF	NF	Unknown
SR6M25	*Comamonas jiangduensis*	NF	-	-	NF	NF	Unknown

[a], Bacterial species were confirmed using the SpeciesFinder-2.0 tool (https://cge.food.dtu.dk/services/SpeciesFinder/).

[b], Antibiotic resistance genes (ARGs) were seek using the following database: ResFinder-4.5.0 (http://genepi.food.dtu.dk/resfinder)* and RGI (Resistance Gene Identifier) from CARD (Comprehensive Antibiotic Resistance Database) platform (https://card.mcmaster.ca/home)**.

[c], In bold, efflux-pump-mediated resistance genes.

[d], Antibiotic and other compounds: A, amphenicols; AC, aminocoumarins; AG, aminoglycosides; B, beta-lactams; D, disinfectants and antiseptics; DP, diaminopyrimidines; F, fosfomicin; M, macrolides; MAR, multiple antibiotic resistance; N, nitroimidazoles; P, polypeptides; Q, quinolones; R, rifamycins; S, sulfonamides; T, tetracyclines.

[e], Plasmids were found using the PlasmidFinder-2.0 too (https://cge.food.dtu.dk/services/PlasmidFinder/).

[f], Virulance genes were screed using the database: VirulenceFinder-2.0 (https://cge.food.dtu.dk/services/VirulenceFinder/).

[g], The multilocus sequence typing were infered by the tools MLST-2.0 tool (https://cge.food.dtu.dk/services/MLST/) and PubMLST (https://pubmlst.org/). The threshold values used for all databases was >90% similarity. NF, not found

References

CGE (Centro de Gerenciamento de Emergências Climáticas). Retrieved from https://www.cgesp.org/v3/ [date visited: 30/5/25].

NMET (Instituto Nacional de Meteorologia). Retrieved from https://portal.inmet.gov.br/ [date visited: 30/5/25].
